# Selections

**Published:** 1895-05

**Authors:** 


### Selections.

### THE VALUE OF PEROXIDE OF HYDROGEN PREPARATIONS.

The following brands have been tested for volume of available oxygen, amount of residue, degree of acidity, and amount of soluble baryta salts contained therein, as per the following table:

| Number. | Brands. | Volume of Available Oxygen, determined by Means of a Solution containing 5.665 Grammes of Permanganate of Potash per Liter of distilled Water. | Residue obtained from 100 c. c. of Peroxide of Hydrogen dried at 120° C. | Acidity expressed in Cubic Centimetres of Normal Volumetric Soda Solution for 100 c. c. of Peroxide. | Baryta found in Soluble Baryta Salts contained in 100 c. c. of Peroxide. |
|---|---|---|---|---|---|
| 1 | John Bene's Peroxide of Hydrogen, Medicinal | 10.50 | 0.1886 | 2.19 | None. |
| 2 | Hydrozone | 27.35 | 0.2180 | 3.11 | None. |
| 3 | Larkin & Scheffer's Peroxide of Hydrogen, Medicinal | 9.65 | 0.1206 | 6.75 | None. |
| 4 | Mallinckrodt's Peroxide of Hydrogen, Medicinal | 9.55 | 0.1408 | 1.43 | None. |
| 5 | Marchand's Peroxide of Hydrogen, Medicinal | 16.55 | 0.5640 | 1.29 | None. |
| 6 | McKesson & Robbins's Peroxide of Hydrogen, Medicinal | 10.95 | 0.0540 | 0.44 | None. |
| 7 | Merck & Co.'s Peroxide of Hydrogen, Medicinal | 0.50 | 0.2418 | 4.57 | None. |
| 8 | Oakland Chemical Co.'s Peroxide of Hydrogen, Medicinal | 10.50 | 0.0382 | 0.34 | 0.0017 |
| 9 | Peuchot's Peroxide of Hydrogen, Medicinal | 10.60 | 0.4674 | 1.77 | 0.0018 |
| 10 | Powers & Weightman's Peroxide of Hydrogen, Medicinal | 8.40 | 0.0830 | 2.03 | None. |
| 11 | Pyrozone, 3 per cent. | 11.20 | 0.0534 | 0.76 | None. |
| 12 | Rosengarten & Sons' Peroxide of Hydrogen, Medicinal | 3.10 | 0.1002 | 0.25 | None. |
| 13 | Smith, Kline & French Co.'s Peroxide of Hydrogen, Medicinal | 6.15 | 0.0880 | 2.60 | None. |
| 14 | E. R. Squibb's Peroxide of Hydrogen, Medicinal | 12.40 | 1.0040 | 12.04 | None. |

By referring to this table it is easily understood that sample No. 2, "Hydrozone," is far superior to any other brand which has ever been made, not only on account of its containing a much larger amount of available oxygen, but also owing to the presence of a small quantity of several essential oils, the respective nature of which could not be determined, very likely because they have been submitted to the oxidizing action of peroxide of hydrogen before being used to make "hydrozone."

I attribute to this small quantity of essential oils the great superiority of hydrozone over any other brands of $H_2O_2$ as a healing agent.

When hydrozone is diluted with distilled water, in the proportion of half and half, the resulting mixture contains about 13.5 volumes of available oxygen, and its bactericide power still remains the same as the bactericide power of sample No. 5, which contains 16.55 volumes of available oxygen.

*Acidity.*—The fourteen brands which I have examined contain free acids (phosphoric, sulphuric, muriatic); and I must say that peroxide of hydrogen, medicinal, should never be made neutral before using, even in the most delicate cases. Neutral peroxide of hydrogen rapidly decomposes under all conditions of exposure.

The keeping properties of $H_2O_2$ solutions vary a great deal with the degree of purity and the percentage of free acids contained therein.

If the proportion of acid is too large, the profession well know that it acts as an irritant upon diseased surfaces. If it is too small, the solution don't keep well.

My opinion is that a standard solution of medicinal $H_2O_2$ must answer the following tests:

1. It should contain at least fifteen volumes of available oxygen.
2. The quantity of free acids contained in one hundred cubic centimetres should require not less than one cubic centimetre and not more than three cubic centimetres of normal volumetric soda solution, to be made neutral. Such a small quantity of free acid is not objectionable.
3. It should not contain any soluble baryta salts.
4. It must be free from sediment.

H. ENDEMANN, PH.D.,
*Chemist.*

NEW YORK.

